# Pulmonary Densitovolumetry Using Computed Tomography in Patients with Nontuberculous Mycobacteria: Correlation with Pulmonary Function Tests

**DOI:** 10.1155/2019/5942783

**Published:** 2019-02-03

**Authors:** Patricia Gomes Cytrangulo De Marca, Telma Goldenberg, Fernanda Carvalho Queiroz Mello, Alysson Roncally Silva Carvalho, Alan Ranieri Medeiros Guimarães, Roberto Mogami, Agnaldo José Lopes

**Affiliations:** ^1^Post-Graduate Program in Medical Sciences, State University of Rio de Janeiro, Rio de Janeiro, Brazil; ^2^Post-Graduate Program in Clinical Medicine, Federal University of Rio de Janeiro, Rio de Janeiro, Brazil; ^3^Laboratory of Respiration Physiology, Carlos Chagas Filho Institute of Biophysics, Federal University of Rio de Janeiro, Rio de Janeiro, Brazil; ^4^Laboratory of Pulmonary Engineering, Biomedical Engineering Program, Alberto Luiz Coimbra Institute of Post-Graduation and Research in Engineering, Federal University of Rio de Janeiro, Rio de Janeiro, Brazil; ^5^Rehabilitation Sciences Post-Graduate Program, Augusto Motta University Center (UNISUAM), Rio de Janeiro, Brazil

## Abstract

**Background:**

Since nontuberculous mycobacterial pulmonary disease (NTM-PD) is a condition with increasing morbidity, a more detailed knowledge of radiological aspects and pulmonary function plays a relevant role in the diagnosis and appropriate therapeutic management of these patients.

**Objectives:**

The purpose of this study was to evaluate changes in lung parenchyma through computed tomography (CT) densitometry and, secondarily, to analyze its correlation with pulmonary function testing (PFT) in patients with NTM-PD.

**Methods:**

This is a cross-sectional study in which 31 patients with NTM-PD and 27 controls matched by sex, age, and body mass index underwent CT pulmonary densitovolumetry and pulmonary function tests including spirometry and body plethysmograph.

**Results:**

Based on the total lung volume (TLV) and total lung mass (TLM) measurements, the cumulative mass ratios were calculated for 3% (M3), 15% (M15), 85% (M85), and 97% (M97) of the TLV. We also calculated the complement, which is represented by TLM (100%) minus the mass of 15% (C85) or 3% (C97) of the TLV. Patients with NTM-PD presented lower values of M3 and M15 than controls, with greater significant differences in the apical third and middle third measurements. Compared to controls, patients with NTM-PD showed higher values of C85 and C97, although significant differences were observed only in the basal third measurements. There were negative correlations of total lung capacity with M3 and M15 in the middle third and apical third measurements. There were positive correlations of residual volume and airway resistance with M3 at the apical third measurement.

**Conclusions:**

Patients with NTM-PD show reduced lung mass and increased lung mass in the apical and basal regions of the lungs, respectively. Furthermore, there is a relationship between lung mass measurements and pulmonary function parameters.

## 1. Introduction

The nontuberculous mycobacteria (NTMs) are increasingly recognized worldwide as an important cause of infection in immunocompetent patients, possibly due to an improvement in the clinical awareness, the increase in the number of susceptible individuals, and technological advances in molecular microbiology [1–3]. NTMs are isolated in environmental sources, such as water and soil, and may be associated with severe lung disease, being an increasing cause of morbidity and mortality and occurring mainly in the elderly, with or without comorbidities. The incidence of this infection is progressively increasing worldwide, although estimates vary between regions [4]. In the United States, the incidence rate is estimated at 7.2/100,000 inhabitants, with 5.6/100,000 inhabitants having pulmonary involvement [5]. Among the several factors that may be contributing to the increase of these infections are the aging of the population with chronic lung diseases and advances in radiological methods that have improved the identification of pulmonary abnormalities [4, 6].

NTM pulmonary disease (NTM-PD) is the most common clinical manifestation of NTM infections, which are most frequently caused by* Mycobacterium avium/intracellulare complex *(MAC),* M. abscessus*,* M. kansasii*,* M. malmoense,* and* M. xenopi *[2, 6–8]. The distribution of species among the various regions of the world is likely to depend both on environmental factors and on patient-related factors, such as comorbidities [9]. Although patients suffering from chronic lung disease are particularly susceptible to NTM-PD, many affected patients have no apparent risk factors [6]. The criteria established to confirm the diagnosis of NTM-PD include clinical data, identification of mycobacteria, and changes in imaging tests [8]. However, diagnosis is generally difficult because clinical manifestations are nonspecific, and bacterial isolation in the sputum or bronchoalveolar lavage may represent only airway colonization [10]. Therefore, imaging exams have a relevant role for the definitive diagnosis of NTM-PD, and high-resolution computed tomography (CT) should be performed in individuals suspected of NTM-PD [1, 4]. Tomographically, NTM-PD can present in two forms: the classic form, with cavities in the upper lobes similar to tuberculosis, and the form in which airway disease predominates, with bronchiectasis and bronchiolitis [11]. A recent study in our country with chest CT in patients with an* M. kansasii* infection showed a predominance of cavities and the pattern of involvement of small and large airways (characterized by bronchiectasis and bronchiole filling) [2].

Using CT, various computer tools for segmenting the chest images have been developed in recent years, including multiplanar reformation, regional lung attenuation analysis, and quantification of anatomical images with area and lung volume measurements [12, 13]. Through quantification of lung volume using multidetector CT (q-MDCT), pulmonary density can be measured by analyzing distribution histograms of pulmonary attenuation values in Hounsfield units [13, 14]. Using quantitative CT analysis in patients with MAC, one study showed that pulmonary infiltration and cavity volume were important determinants for the poor performance of these patients in pulmonary function testing (PFT) [15]. However, to the best of our knowledge, no study has evaluated q-MDCT in patients with other NTMs or the impact of these findings on PFT.

The importance of new diagnostic modalities to optimize NTM-PD management has been increasingly discussed [4]. Since NTM-PD is a condition with increasing morbidity, a more detailed knowledge of radiological aspects and pulmonary function plays a relevant role in the diagnosis and appropriate therapeutic management of these patients. Thus, the objective of this study was to evaluate changes in the lung parenchyma through CT densitovolumetry and to analyze its correlation with the parameters provided by the PFT in patients with NTM-PD.

## 2. Materials and Methods

### 2.1. Patients

In the period between May 2017 and January 2018, a cross-sectional study was conducted with 48 patients with NTM-PD aged ≥18 years recruited at the Professor Hélio Fraga Reference Center of the National School of Public Health Sergio Arouca/Oswaldo Cruz Foundation, Rio de Janeiro, RJ, Brazil. All patients with NTM-PD enrolled into the study at the time of being given a new diagnosis of NTM-PD. The diagnosis of NTM-PD was made by combining clinical, radiological, and microbiological criteria, as recommended by the American Thoracic Society (ATS)/Infectious Disease Society of America (IDSA) [8]. Each respiratory sample was collected under sterile conditions and processed immediately or conserved at 4°C. Smears were stained using the auramine-phenol acid-fast method. All cultures were grown in both solid Löwenstein-Jensen medium and the BACTEC MGIT 960 system. NTM species were identified using sequence analysis of the 16S rRNA gene by the algorithm descried in the Clinical and Laboratory Standards Institute (CLSI) guidelines [16]. Drug susceptibility testing and reporting followed the recommendations of the CLSI [16].

We used the following exclusion criteria: history of pleuropulmonary tuberculosis (n=6); positive serology for HIV (n=3); coinfection with other pathogens including invasive mold disease or pseudomonas (n=3); previous smoking history of more than 10 pack-years (n=2); inability to perform PFT (n=2); and current smoking report (n=1). Thus, the sample evaluated consisted of 31 patients, 11 of them using antimicrobials prior to the CT scan or PFTs.

We also evaluated a control group that was matched by sex, age, and body mass index (BMI) and composed of subjects who, after having already performed chest CT scan for some other reason, were invited to perform the PFT. This group consisted of 27 subjects aged ≥18 years with a chest CT with no abnormalities. Subjects in this group who reported previous pleuropulmonary disease (n=3) or smoking history (previous or current) of more than 10 pack-years (n=3) were excluded from the study.

For each study participant (patients or controls), PFTs and CT scan were performed with a maximum interval of one month. All subjects signed the informed consent form, and the protocol was approved by the Research Ethics Committee of the Federal University of Rio de Janeiro under approval number CAAE-59459216.1.1001.5257.

### 2.2. Pulmonary Function Testing

Pulmonary function tests consisted of spirometry and body plethysmograph. Measurements were made through an HD CPL (nSpire Health, Inc., Longmont, CO, USA), following the standard requirements for performing and interpreting the exams [17]. The results of lung function tests were expressed as a percentage of the predicted values, using established equations for our population [18, 19].

### 2.3. Computed Tomography and Densitovolumetry

CT scans were performed in a 64-channel, multislice Philips CT system (Brilliance 40, Philips Medical Systems, Cleveland, OH, USA). The acquisitions were performed in the axial plane with the patients in the dorsal decubitus position using the following parameters: 120 kV, 458 mA (which varied according to the biotype of the patient), slice thickness of 2 mm, and pitch of 2 mm from the jugular notch to the xiphoid process at maximal inspiration, without administration of intravenous contrast. Abnormalities in chest CT scans were interpreted by two independent readers (P.G.C.M. and R.M., with 7 years and 15 years of experience in examinations of the chest, respectively), who were blinded for clinical data and PFT exams. Disagreements were resolved by consensus after a joint reassessment of CT scans.

Segmentation of the lung parenchyma was performed, and the images were later exported to a software program (CT-Processing) written in MATLAB (MathWorks, Natick, MA, USA). After the end of this process, pulmonary densitovolumetry could be performed using q-MDCT.

Total lung volume (TLV) and total lung mass (TLM) were calculated considering only the image inside the region of interest (lung parenchyma). The TLV (i.e., the sum of air plus tissue volume) was calculated as follows: ([size of the pixel]^2^  × slice thickness × total number of pixels of the region of interest for the whole lung). Mass of the lungs was calculated as follows: ([1 – voxel density/-1000] × [size of the pixel]^2^  × slice thickness × total number of pixels of the region of interest for the whole lung) [12, 20].

#### 2.3.1. Lung Mass Calculation

Before lung mass calculation, the average densities of air inside the trachea (HU_Air_) and blood in the descending aorta (HU_Tissue_) were measured. The intensity values of all voxels in lung parenchyma were then linearly rescaled considering that HU_Air_ and HU_Tissue_ should be equal to -1000 and +50 Hounsfield units (HU), respectively [21, 22]. Assuming that any region of the lung can be modeled as a mixture of tissue (cells, blood cells, collagen, elastin, and so forth) and air, the density in such a region, in HU, can be expressed as the average of air and tissue densities, weighed by their volume fractions in that region. The mass of each voxel, in grams, in the previously segmented lung parenchyma was then calculated as follows: mass (g) = [HU_Voxel_ - HU_Air_ / HU_Tissue_ - HU_Air_] × voxel volume × 1.04 g/mL, where 1.04 mg/mL is the lung tissue density and HU_Voxel_ is the respective voxel density in HU [22].

The cumulative lung mass (M) was calculated as follows: M_(k)_ = Σ^+50/*k*=-1000^voxel mass_(k)_ × number of voxels with a given HU_(k)_ + M_(k-1)_. For k equal to 1, M_(k-1)_ will be equal to zero. By definition, M_(k)_ is equal to 0 g for HU_Voxel_ <-1000 HU and equal to the voxel volume, in mL, multiplied by 1.04 g/mL for HU_Voxel_ >+50 HU.

Based on the TLV and TLM measurements, the cumulative mass ratios were calculated at 3% (M3), 15% (M15), 85% (M85), and 97% (M97) of the TLV. These values are pretty close to ±2 standard deviations (SD) and ±1 SD around the mean mass. In addition, we also calculated the complement, which is represented by TLM (100%) minus the mass of 15% (C85) or 3% (C97) of the TLV.

### 2.4. Statistical Analyses

Considering the association between the CT pulmonary densitovolumetry and the PFT variables as the main outcome of this study, a minimal sample size of 30 participants was necessary to observe a minimal correlation of 0.41 (weak or higher) at a 5% significance level and 80% study power.

Comparison of clinical variables, PFT, and CT densitovolumetry among patients with NTM-PD and control subjects was assessed by Student's t-test for independent samples for numerical data and by Fisher's exact test for categorical data. The Pearson correlation coefficient (r) was calculated to investigate the associations between the CT densitovolumetry variables and the PFT parameters. Data analysis was performed using software SAS 6.11 (SAS Institute, Inc., Cary, NC, USA). The level of statistical significance was set at* p*<0.05.

## 3. Results

Of the 31 patients with NTM-PD who comprised the study sample, 15 (48.4%) were men. The mean age of the patients with NTM-PD was 57.2 ± 16.5 years. Seven patients had a previous history of smoking, with a smoking load of ≤10 pack-years. In most of our patients (58.1%),* M. kansasii* was responsible for lung disease. After* M. kansasii*, the second most common was* M. avium* complex (25.8%).

Regarding the controls, patients with NTM-PD showed lower values of forced vital capacity (FVC, 82.7 ± 25.3 vs. 102 ± 8.12 % predicted,* p*=0.005) and forced expiratory volume in one second (FEV_1_, 72.7 ± 25.4 versus 101.2 ± 8.75 % predicted,* p*=0.003). However, patients with NTM-PD showed higher values of residual volume (RV, 119 ± 45.6 versus 75.8 ± 23.2 % predicted,* p*=0.001), RV/total lung capacity (RV/TLC, 37.5 ± 12.4 versus 25.7 ± 8.90 %,* p*=0.02), and airway resistance (Raw, 6.53 ± 3.77 versus 2.31 ± 2.13 cmH_2_O/L/s,* p*<0.001). Clinical data and outcomes of the patients' and controls' PFTs are shown in Table 1.

In CT scans, all patients showed characteristic signs of lung disease in at least one lobe. The main alterations found in CT examinations were as follows: architectural distortion (80.6%), reticular opacities (77.4%), bronchiectasis (71%), cavities (64.5%), centrilobular nodules (61.3%), atelectasis (51.6%), small consolidations (48.4%), large consolidations (32.2%), and subpleural blebs and emphysema (25.8%). Bronchiectasis and cavities predominated in the upper lobes in 61.3% of cases, whereas reticular opacities and centrilobular nodules showed a more heterogeneous distribution, with involvement of the lower lobes in 41.9% of the cases. Subpleural blebs and emphysema predominated in the upper lobes in 90.3% of cases.

**Figure 1:**
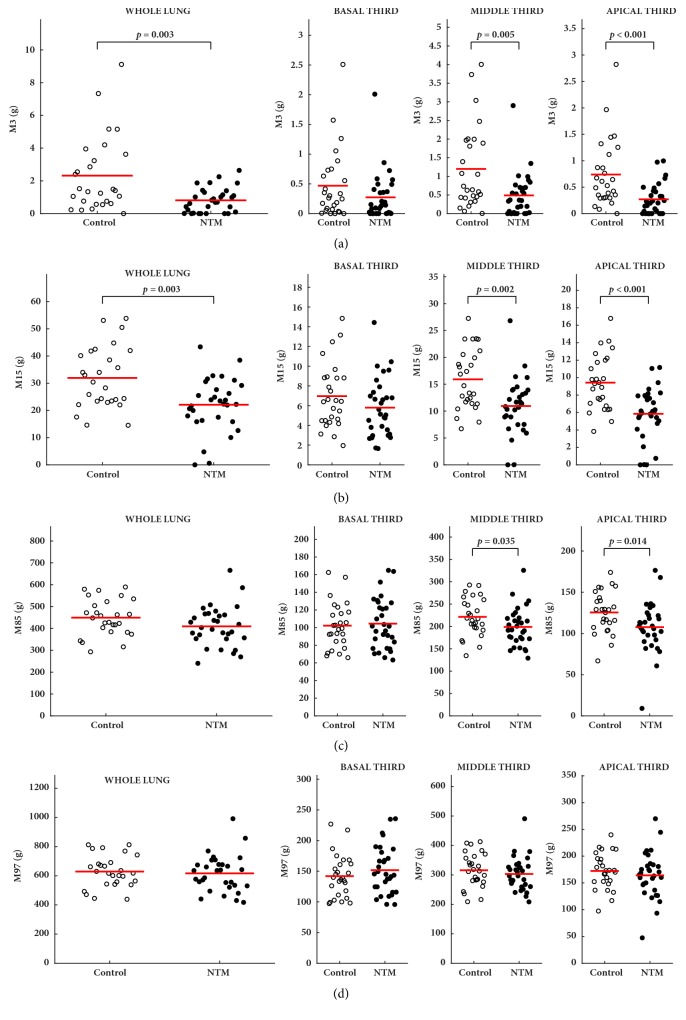
Comparisons between the control group and the nontuberculous mycobacteria group (NTM) for the measures of cumulative mass relations in 3% (M3) (a), 15% (M15) (b), 85% (M85) (c), and 97% (M97) (d) of the total lung volume.

**Figure 2:**
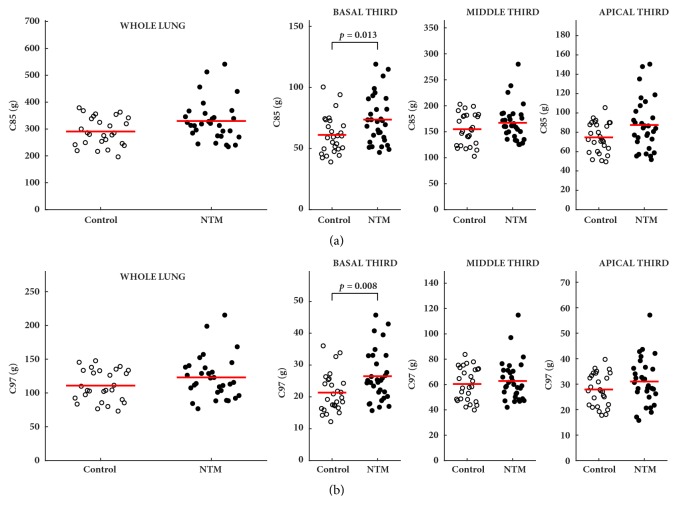
Comparisons between the control group and the nontuberculous mycobacteria group (NTM) for complement measurements, which represents the total lung mass (100%) minus the 15% (C85) (a) or 3% (C97) (b) mass of the total lung volume.

In CT pulmonary densitovolumetry, NTM-PD patients had lower M3 values than controls, with significant differences for whole lung measurements (0.81 ± 0.25 versus 2.42 ± 0.65 g,* p*=0.003); these statistically significant differences were in apical third measurements (0.25 ± 0.11 versus 0.76 ± 0.23 g,* p*<0.001) and, to a lesser extent, in middle third measurements (0.51 ± 0.21 versus 1.24 ± 0.36 g,* p*=0.005). In addition, NTM-PD patients had lower M15 values than controls, with significant differences for whole lung measurements (21.8 ± 4.70 versus 32.3 ± 5.74 g,* p*=0.003); these statistically significant differences were more evident in apical third measurement (5.93 ± 1.44 versus 9.49 ± 2.01 g,* p*<0.001) and, to a lesser extent, in middle third measurements (11 ± 2.33 versus 16.7 ± 3.04 g,* p*=0.003) (Figure 1). Compared to controls, patients with NTM-PD showed higher values of C85 and C97, although significant differences were observed only in basal third measurements: 76 ± 9.37 versus 61.1 ± 8.45 g,* p*=0.013 and 26.2 ± 4.83 versus 21.8 ± 3.96 g,* p*=0.008, in C85 and C97, respectively (Figure 2).

We also evaluated correlations between the CT pulmonary densitovolumetry findings and the PFT parameters. FVC (L) was negatively correlated with M3 in the apical third (r=-0.42;* P*=.021) and M15 in the apical third (r=-0.37;* p*=0.047). TLC was negatively correlated with the following measurements: M3 in the whole lung (r=-0.66;* p*<0.001); M3 in the middle third (r=-0.60;* p*<0.001); M3 in the apical third (r=-0.72;* p*<0.001); M15 in the whole lung (r=0.66;* p*<0.001); and M15 in the apical third (r=-0.50;* p*=0.005). RV (L) and Raw cmH_2_O/L/s were positively correlated with M3 in the apical third (r=0.48;* p*=0.007; r=0.41;* p*=0.026, respectively).

## 4. Discussion

The main findings in the present study were that patients with NTM-PD, when compared to controls, showed reductions in lung mass, which were more pronounced in the apical and middle thirds of the lungs. At the lung bases, however, patients with NTM-PD exhibited increased lung mass compared to controls. Functionally, there was a reduction of pulmonary volumes in relation to the controls, although functional alterations compatible with airflow reduction were also noted. Moreover, greater structural damage resulted in greater functional changes. To the best of our knowledge, this is the first study to evaluate the lung parenchyma of NTM-PD patients by quantification of lung mass and its correlation with measures of respiratory function.

Similar to the Brazilian studies by de Mello et al. [23] and Mogami et al. [2], we observed that* M. kansasii* was the most frequent NTM in our sample. However, the high geographical variability of* M. kansasii*, which has a high prevalence in Western Europe and lower prevalence in countries such as the United States and Australia, should be highlighted [24, 25]. In our study, patients with a prior history of pleuropulmonary tuberculosis—which has a high incidence rate in Brazil [23]—were excluded. This is important since once a previous history of pleuropulmonary tuberculosis can adversely impact radiological and functional findings [7].

In NTM-PD, an imaging study is essential to establish the diagnosis and to assist in the clinical management of the disease [7, 8, 26]. As NTM-PD symptoms cannot be differentiated from those of other respiratory diseases, and culture examination takes 2-3 weeks for most NTM to grow, NTM-PD is generally initially suspected by radiological findings [27]. In our study, we observed a high frequency of bronchiectasis and cavities, which can be at least partly explained by the higher prevalence of* M. kansasii*. In line with our findings, several studies have also reported bronchiectasis and cavities as common findings in patients with* M. kansasii* pulmonary infection, predominantly in the upper lobes, either through simple X-ray or through chest CT [2, 28, 29]. To elucidate the accuracy and interobserver agreement for the diagnosis of NTM-PD based on the findings of chest CT, Kwak et al. [27] showed that the tree-in-bud pattern, consolidation, and atelectasis are associated with correct diagnosis of NTM-PD, while the presence of pleural effusion leads to false diagnosis. In fact, in our study population, these three findings (together or in combination) were also present in a significant portion of CT scans. Notably, in our sample, a high frequency of architectural distortion was observed that can be explained by NTM-PD itself with excavations and bronchiectasis that lead to atelectasis of the lobe [2].

Through CT pulmonary densitovolumetry, we observed a reduction in lung mass in patients with NTM-PD compared to controls, and this reduction was more accentuated in the measurements of the apical third and, to a lesser extent, in the measurements of the middle third. The reduction of lung mass in patients with NTM-PD can be explained by the greater destruction of the lung parenchyma in the upper two-thirds of the lungs, especially because of the presence of cavitary and/or bronchiectasis lesions [3, 27]. On the other hand, our findings also showed that these patients presented an increase in lung mass in the basal third, as demonstrated by the measurements of C85 and C97. The greater mass in the lung bases of the NTM-PD group can be justified by findings compatible with fibrosis (reticular opacities) and airway filling (tree-in-bud pattern), which were frequently seen in the lower lobes in our sample. In addition, the presence of a lower ventilation-perfusion ratio in the lung bases due to the compensatory increase in the remaining pulmonary circulation in this area due to the important involvement of the lung parenchyma in the upper thirds of the lungs is also a possible explanation for this finding [30]. Also worth mentioning is that, more recently, chest CT examination has been recommended shortly before starting treatment and at the end of treatment for NTM in order to document the radiological response [1]. More frequent radiological monitoring may be indicated in selected individuals during the course of treatment for NTM-PD. In this context, we believe that CT pulmonary densitovolumetry may be a useful tool in the management of NTM-PD. Some advantages of CT pulmonary densitovolumetry are the greater detailing of lung lesions and their good correlation with lung function. In addition, it can be used to target bronchial wash in individual cases. In fact, a chest CT scan followed by CT-directed bronchial wash after 6 and 12 months of therapy has been recently recommended in the evaluation of the microbiological response in patients who are unable to expectorate sputum [1, 31].

As expected, our patients showed important fibrosing changes in the lungs that functionally affected lung volume reduction. Similarly, Park et al. [32] have shown, in a prospective cohort of NTM-PD patients, a rapid decline in lung function that was most evident in those who did not respond to treatment. However, we also observed functional alterations compatible with airflow reduction, with elevation of RV, RV/TLC, and Raw, which may not be explained by the effect of smoking in our sample since we exclude those with previous smoking history of more than 10 pack-years. This cut-off point (>10 pack-years) has been used to define smoking load that can impact lung function [33, 34]. Interestingly, Huang et al. [35] showed that, after controlling for confounding factors, patients with chronic obstructive pulmonary disease and NTM-PD had a greater decline in lung function than those without NTM-PD.

The correlation between imaging and pulmonary function is an important step in the evaluation of NTM-PD since the therapeutic response is almost always evaluated using clinical data and PFT results [9]. In the present study, we observed negative correlations between pulmonary mass measurements and volume parameters assessed by PFT. Interestingly, the greatest number of correlations and the strongest correlations were those with mass measurements in the apical third of the lungs, which reflects the impact of the structural damage of that region on the lung volumes. In addition, we observed that functional measures that reflect airflow limitation (RV and Raw) positively correlated with mass measurements in the apical third of the lungs, showing the impact of pulmonary blebs and emphysematous lesions located in this area on the respiratory function measurements.

In a recent study, Asakura et al. [15] evaluated patients with MAC through q-MDCT and observed that the TLV and the mean value of CT were significantly correlated with all the results of the PFT. In that same study, there was a significant correlation between the proportion of pulmonary infiltration and the PFT results, especially FVC (r_s_=-0.52), RV (r_s_=-0.51), and TLC (r_s_=-0.59); the cavity volume was strongly correlated with FVC (r_s_=-0.78) in patients with cavity, while the proportion of pulmonary infiltration was strongly correlated with FVC (r_s_=-0.53) in patients without cavity. Certain NTM species, such as* M. kansasii*, are often considered more virulent than other species, and, therefore, a higher prevalence of* M. kansasii* in our sample may at least partly justify the differences in the observed results in the correlations between the densitovolumetry and PFT in relation to those observed by Asakura et al. [15].

This study had some limitations. First, it was a single-center study. Second, we did not perform subgroup analyses according to each NTM species because of the small number of patients in each group. This is important because there may be differences in imaging and pulmonary function among NTM-PD patients infected with different species. Third, we did not perform an expiratory CT study for airway involvement, and, therefore, the obstruction of the small airways may have been underestimated. Despite these limitations, we believe CT pulmonary densitovolumetry may be an auxiliary tool in the follow-up of patients with NTM-PD. Thus, further investigations of the technique in this population may be important, including the longitudinal changes that occur in response to treatment.

In summary, our results show that patients with NTM-PD have reductions in lung mass, especially in the more apical regions of the lungs. However, in the more basal regions of the lungs, there is a slight accumulation of lung mass. Despite the restrictive functional damage with reduction in lung volumes, functional signs of airflow limitation are also present in these patients. Furthermore, there is a relationship between lung mass measurements and pulmonary function parameters.

## Figures and Tables

**Table 1:** Patients and control subjects characteristics.

	**NTM Group**	**Control Group**	***p***
**No. of subjects**	31	27	
**Demographic data**			
Males, %	15 (48.4%)	13 (48.1)	0.92*∗*
Age, y	57.2 ± 16.5	52.4 ± 15.3	0.12†
BMI, kg/m^2^	23.8 ± 4.87	25.8 ± 4.72	0.16†
**NTM species**			
*M. kansasii*	18 (58.1)		
*M. avium* complex	8 (25.8)		
*M. fortuitum*	3 (9.68)		
*M. gordonae*	1 (3.23)		
*M. abscessus*	1 (3.23)		
**Lung function**			
FVC (% predicted)	82.7 ± 25.3	102 ± 8.12	**0.005**†
FEV_1_ (% predicted)	72.7 ± 25.4	101.2 ± 8.75	**0.003**†
FEV_1_/FVC (%)	84.3 ± 15.2	82.1 ± 5.28	0.34†
TLC (% predicted)	90.4 ± 18.5	94.6 ± 9.11	0.25†
RV (% predicted)	119 ± 45.6	75.8 ± 23.2	**0.001**†
RV/TLC (%)	37.5 ± 12.4	25.7 ± 8.90	**0.02**†
Raw (cmH_2_O/L/s)	6.53 ± 3.77	2.31 ± 2.13	**<0.001**†

Values are means ± SD or number (%). *∗*Fisher exact test. †Student *t-*test.

BMI = body mass index; NTM = *nontuberculous mycobacteria*; FVC = forced vital capacity; FEV_1_ = forced expiratory volume in one second; TLC = total lung capacity; RV = residual volume; Raw = airway resistance.

## Data Availability

The chest CT scans, pulmonary function tests, and patients' data used to support the findings of this study are available from the corresponding author upon request.

## Abbreviations

ATS: American Thoracic Society
C85: Complement represented by total lung mass minus the mass of 15% of the total lung volume
C97: Complement represented by total lung mass minus the mass of 3% of the total lung volume
CT: Computed tomography
FEV_1_: Forced expiratory volume in one second
FVC: Forced vital capacity
HU: Hounsfield units
HU_Air_: Average density of air inside the trachea
HU_Tissue_: Average density of blood in the descending aorta
HU_Voxel_: Voxel density in Hounsfield unit
IDSA: Infectious Disease Society of America
M: Cumulative lung mass
M3: Cumulative mass ratio calculated at 3% of the total lung volume
M15: Cumulative mass ratio calculated at 15% of the total lung volume
M85: Cumulative mass ratio calculated at 85% of the total lung volume
M97: Cumulative mass ratio calculated at 97% of the total lung volume
MAC: * Mycobacterium avium/intracellulare complex*
NTM: Nontuberculous mycobacteria
NTM-PD: Nontuberculous mycobacteria pulmonary disease
PFT: Pulmonary function testing
q-MDCT: Quantification of lung volume using multidetector computed tomography
Raw: Airway resistance
RV: Residual volume
TLC: Total lung capacity
TLM: Total lung mass
TLV: Total lung volume.

## References

[1] Haworth C. S., Banks J., Capstick T. (2017). British Thoracic Society Guideline for the management of non-tuberculous mycobacterial pulmonary disease (NTM-PD). *BMJ Open Respiratory Research*.

[2] Mogami R., Goldenberg T., de Marca P. G. C., Mello F. C. D. Q., Lopes A. J. (2016). Pulmonary infection caused by Mycobacterium kansasii: Findings on computed tomography of the chest. *Radiologia Brasileira*.

[3] Chu H.-Q., Li B., Zhao L. (2015). Chest imaging comparison between non-tuberculous and tuberculosis mycobacteria in sputum acid fast bacilli smear-positive patients. *European Review for Medical and Pharmacological Sciences*.

[4] Stout J. E., Koh W.-J., Yew W. W. (2016). Update on pulmonary disease due to non-tuberculous mycobacteria. *International Journal of Infectious Diseases*.

[5] Cassidy P. M., Hedberg K., Saulson A., McNelly E., Winthrop K. L. (2009). Nontuberculous mycobacterial disease prevalence and risk factors: a changing epidemiology. *Clinical Infectious Diseases*.

[6] Wassilew N., Hoffmann H., Andrejak C., Lange C. (2016). Pulmonary disease caused by non-tuberculous mycobacteria. *Respiration*.

[7] Kim C., Park S. H., Oh S. Y. (2017). Comparison of chest CT findings in nontuberculous mycobacterial diseases vs. Mycobacterium tuberculosis lung disease in HIV-negative patients with cavities. *PLoS ONE*.

[8] Griffith D. E., Aksamit T., Brown-Elliott B. A. (2007). An official ATS/IDSA statement: diagnosis, treatment, and prevention of nontuberculous mycobacterial diseases. *American Journal of Respiratory and Critical Care Medicine*.

[9] Rawson T. M., Abbara A., Kranzer K. (2016). Factors which influence treatment initiation for pulmonary non-tuberculous mycobacterium infection in HIV negative patients; a multicentre observational study. *Respiratory Medicine*.

[10] Barreto M. M., Rodrigues R. S. (2016). The importance of computed tomography of the chest in cases of suspected infection with nontuberculous mycobacteria (Mycobacterium kansasii). *Radiologia Brasileira*.

[11] Rubin S. A. (1997). Tuberculosis and atypical mycobacterial infections in the 1990s. *RadioGraphics*.

[12] Lopes A. J., Mogami R., Camilo G. B., Machado D. C., Melo P. L., Carvalho A. R. S. (2015). Relationships between the pulmonary densitometry values obtained by CT and the forced oscillation technique parameters in patients with silicosis. *British Journal of Radiology*.

[13] Camilo G. B., Carvalho A. R. S., Guimarães A. R. M. (2017). Computed tomography airway lumen volumetry in patients with acromegaly: Association with growth hormone levels and lung function. *Journal of Medical Imaging and Radiation Oncology*.

[14] Wielpütz M. O., Weinheimer O., Eichinger M. (2013). Pulmonary emphysema in cystic fibrosis detected by densitometry on chest multidetector computed tomography.. *PLoS ONE*.

[15] Asakura T., Yamada Y., Namkoong H. (2017). Impact of cavity and infiltration on pulmonary function and health-related quality of life in pulmonary Mycobacterium avium complex disease: A 3-dimensional computed tomographic analysis. *Respiratory Medicine*.

[16] (2011). *Susceptibility Testing of Mycobacteria, Nocardia, and Other Aerobic Actinomycetes*.

[17] Culver B. H., Graham B. L., Coates A. L. (2017). Recommendations for a standardized pulmonary function report. an official american thoracic society technical statement. *American Journal of Respiratory and Critical Care Medicine*.

[18] De Castro Pereira C. A., Sato T., Rodrigues S. C. (2007). New reference values for forced spirometry in white adults in Brazil. *Jornal Brasileiro de Pneumologia*.

[19] Neder J. A., Andreoni S., Castelo-Filho A., Nery L. E. (1999). Reference values for lung function tests. I. Static volumes. *Brazilian Journal of Medical and Biological Research*.

[20] Camilo G. B., Carvalho A. R. S., Machado D. C., Mogami R., Melo P. L., Lopes A. J. (2015). CT pulmonary densitovolumetry in patients with acromegaly: A comparison between active disease and controlled disease. *British Journal of Radiology*.

[21] Stoel B. C., Vrooman H. A., Stolk J., Reiber J. H. C. (1999). Sources of error in lung densitometry with CT. *Investigative Radiology*.

[22] Staring M., Bakker M. E., Stolk J., Shamonin D. P., Reiber J. H., Stoel B. C. (2014). Towards local progression estimation of pulmonary emphysema using CT. *Medical Physics*.

[23] de Mello K. G. C., Queiroz Mello F. C., Borga L. (2013). Clinical and therapeutic features of pulmonary nontuberculous mycobacterial disease, Brazil, 1993-2011. *Emerging Infectious Diseases*.

[24] Prevots D. R., Shaw P. A., Strickland D. (2010). Nontuberculous mycobacterial lung disease prevalence at four integrated health care delivery systems. *American Journal of Respiratory and Critical Care Medicine*.

[25] Moore J. E., Kruijshaar M. E., Ormerod L. P., Drobniewski F., Abubakar I. (2010). Increasing reports of non-tuberculous mycobacteria in England, Wales and Northern Ireland, 1995–2006. *BMC Public Health*.

[26] Zheng C., Fanta C. H. (2013). Non-tuberculous mycobacterial pulmonary infection in the immunocompetent host. *QJM: An International Journal of Medicine*.

[27] Kwak N., Lee C. H., Lee H.-J. (2016). Non-tuberculous mycobacterial lung disease: diagnosis based on computed tomography of the chest. *European Radiology*.

[28] Shitrit D., Baum G. L., Priess R. (2006). Pulmonary Mycobacterium kansasii infection in Israel, 1999-2004: Clinical features, drug susceptibility, and outcome. *CHEST*.

[29] Matveychuk A., Fuks L., Priess R., Hahim I., Shitrit D. (2012). Clinical and radiological features of *Mycobacterium kansasii* and other NTM infections. *Respiratory Medicine*.

[30] Mottram C. D. (2013). *Ruppel’s Manual of Pulmonary Function Testing*.

[31] Watanabe K., Shinkai M., Shinoda M., Kaneko T. (2018). Bronchial wash culture is less valuable in patients suspected to have nontuberculous mycobacteria lung disease for bilateral bronchiectasis with nodules. *International Journal of Mycobacteriology*.

[32] Park H. Y., Jeong B.-H., Chon H. R., Jeon K., Daley C. L., Koh W.-J. (2016). Lung function decline according to clinical course in nontuberculous mycobacterial lung disease. *CHEST*.

[33] Lopes A. J., Mafort T. T. (2014). Correlations between small airway function, ventilation distribution, and functional exercise capacity in COPD patients. *Lung*.

[34] Timmins S. C., Diba C., Farrow C. E. (2012). The relationship between airflow obstruction, emphysema extent, and small airways function in COPD. *CHEST*.

[35] Huang C.-T., Tsai Y.-J., Wu H.-D. (2012). Impact of non-tuberculous mycobacteria on pulmonary function decline in chronic obstructive pulmonary disease. *The International Journal of Tuberculosis and Lung Disease*.

